# Supplementary material for: Assessment of the Photosynthetic Apparatus Functions by Chlorophyll Fluorescence and P700 Absorbance in C3 and C4 Plants under Physiological Conditions and under Salt Stress
Source: Int J Mol Sci. 2022 Mar 29;23(7):3768. doi: 10.3390/ijms23073768 (PMC8998893; doi:10.3390/ijms23073768)
Supplement: Supplementary file 1 [file ijms-23-03768-s001.zip › ijms-1638240-supplementary.pdf]

Supplementary Materials

Table S1: Impact of different NaCl concentrations on the chlorophyll content and chlorophyll a/b ratio in pea and maize (uppercase for pea and lower case are for maize).

	Chl a+b (μg/g DW)	Chl a/b
pea		
control	30825±237^{A}	3.22±0.03^{D}
50 mM NaCl	24255±64^{B}	3.47±0.04^{C}
150 mM NaCl	18855±32^{C}	3.88±0.03^{B}
200 mM NaCl	11061±15^{D}	4.07±0.05^{A}
maize		
control	26319±48^{a}	4.49±0.03^{c}
50 mM NaCl	26283±268^{a}	4.55±0.04^{bc}
150 mM NaCl	22360±36^{b}	4.68±0.04^{ab}
200 mM NaCl	15032±19^{c}	4.75±0.03^{a}
